# Multilevel and multicomponent intervention to promote colorectal cancer screening among underserved Vietnamese Americans: A cluster randomized trial

**DOI:** 10.21203/rs.3.rs-3934937/v1

**Published:** 2024-02-08

**Authors:** Grace X. Ma, Lin Zhu, Yin Tan, Phuong Do, Guercie Guerrier, Min Qi Wang, Minhhuyen Nguyen, Tam Tran, Philip Pham

**Affiliations:** 1 Center for Asian Health, Lewis Katz School of Medicine, Temple University, Philadelphia, PA; 2 Department of Urban Health and Population Science, Lewis Katz School of Medicine, Temple University, Philadelphia, PA; 3 University of Maryland School of Public Health, College Park, MD; 4 Department of Medicine, Section of Gastroenterology, Fox Chase Cancer Center, Temple University Health System, Philadelphia, PA; 5 Asian American Buddhist Association, Philadelphia, PA; 6 Vietnamese International Baptist Church of Philadelphia, Philadelphia, PA

**Keywords:** Fecal immunochemical Test (FIT), Culturally-tailored multilevel intervention, Colorectal cancer screening, Underserved populations, Vietnamese Americans

## Abstract

**Purpose:**

The fecal immunochemical test (FIT) is a non-invasive method for colorectal cancer (CRC) screening, particularly effective in underserved Vietnamese American communities with low screening rates. This study reports on a culturally tailored multilevel intervention, incorporating FIT, aimed at increasing CRC screening among these populations aged 50 or above in the Greater Philadelphia metropolitan area.

**Methods:**

From 2017 to 2020, we conducted a two-arm cluster randomized controlled trial to test the efficacy of a culturally tailored, multicomponent multilevel intervention aimed at increasing CRC screening uptake via enhanced self-awareness and self-efficacy, improved access to care, and changes in social norms and removal of stigma. The intervention group received multicomponent, multilevel CRC intervention including provision of a FIT self-sampling kit, with intervention approaches informed by the Centers for Disease Control’s Clinical Preventive Services (CPS) Guidelines for adults 50+. The control group received only the CPS education.

**Results:**

The study sample consisted of 746 eligible Vietnamese American participants recruited from 20 community-based organizations, with 95% having limited English proficiency. At 12-month follow-up, the intervention group showed substantially higher rates of FIT completion (89.56% vs. 7.59%, p < .001) and any CRC testing (91.48% vs. 42.41%, p < .001) compared to the control group.

**Conclusion:**

The results suggest that the community-based, culturally-tailored multilevel intervention, which incorporates with FIT self-testing, effectively enhances CRC screening among low-income Vietnamese Americans. Additionally, these results underscore the significance of community-oriented strategies, like collaborating with relevant community-based organizations, in achieving CRC screening targets.

## Introduction

In the United States, excluding skin cancer, colorectal cancer (CRC) is the third most common malignancy, and second in cancer deaths[1]. The majority of CRC cases, however, can be prevented with proper screening, early detection, and removal of adenomatous polyps[2]. In addition, survival rates is significantly better when CRC is detected through screening before it has spread outside the colon or rectum, compared to when it is detected at later stages [3]. The US Preventive Services Task Force (USPSTF) recommends that adults aged 45 to 75 receive CRC screening[4]. Screening methods includes (1) visual exams, such as colonoscopy, computed tomography (CT) colonography, and flexible sigmoidoscopy; and (2) stool-based tests, such as the guaiac-based fecal occult blood test (gFOBT), the fecal immunochemical test (FIT), and the fecal occult blood test. Non-invasive tests such as FIT are notably accessible and affordable in low-income, racial/ethnic minority communities [5].

Vietnamese Americans are disproportionately affected by CRC, with rates increasing from 35.6 to 46.3 per 100,000 men and from 30.5 to 34.2 per 100,000 women between 1990–1994 and 2009–2011, according to Surveillance, Epidemiology and End Results (SEER) program[6]. Despite this, CRC screening uptake remained suboptimal in this group[7], with only 33% to 61% [8–10] of eligible Vietnamese Americans are up-to-date with CRC screening, which is lower than the 68.8% screening rate in the general US population [11] as well as the 74.4% goal set forth in the Healthy People 2030 initiative [12].

Interventions aimed at increasing CRC screening uptake in Vietnamese Americans primarily focused on addressing individual-level barriers. These include enhancing knowledge about CRC risks and screening methods, raising awareness of CRC screening resources, and providing logistic assistance to CRC screening [13–17]. However, increasing evidence underscores the need for multilevel interventions that target risk factors, barriers or social determinants of health on more than one level, including individual, interpersonal, community, healthcare provider, and health system levels [18, 19]. Among studies that have reached beyond the individual level, two studies have incorporated an interpersonal-level component by using lay health workers or young adult family advocates to facilitate group discussion on CRC screening among family group chats on WhatsApp or Facebook Messenger [14, 20]. One study adopted a community-level approach by distributing culturally tailored educational comic strips at local Vietnamese grocery stores, through which the researchers hoped to promote community-level awareness and to remove stigmas associated with CRC and screening [21].

To our best knowledge, this study represents the first multilevel intervention promoting FIT-based CRC self-screening in low-income Vietnamese Americans. Utilizing a community-based participatory research (CBPR) approach, we developed a culturally tailored, multicomponent intervention addressing specific barriers faced by this group. The goal of the study was to evaluate the effectiveness of this novel intervention, with participant self-screening via FIT as a primary outcome.

## Methods

From 2017 to 2020, we conducted a two-arm cluster randomized controlled trial in the Greater Philadelphia metropolitan area to test the efficacy of a culturally tailored, multicomponent multilevel intervention aimed at increasing CRC screening via FIT among Vietnamese Americans over 50, particularly those with low income. The intervention was designed to address individual-, interpersonal-, and organizational/community-level barriers that Vietnamese Americans face in accessing CRC screening and related healthcare services.

### Study Design

We conducted outreach and enrolled 20 Vietnamese community-based organizations (CBOs) in the Greater Philadelphia metropolitan area, which includes Philadelphia and Southern New Jersey. All 20 CBOs consented in our study. We randomly assigned 10 CBOs to the intervention group and the other 10 to the control group. We engaged collaborating CBO leaders and designated study team members in training about IRB, human subject protection, participant eligibility screening and study protocols. Our research team and the enrolled CBOs jointly conducted eligibility screening and assigned eligible participants to either the intervention or the control group, depending on the study arm assignment of their affiliated CBO sites. We assessed community members for eligibility on the basis of the following inclusion criteria:

1. identify as being of Vietnamese descent
2. at least 50 years old
3. accessible by cell phone
4. no personal history of CRC or colon polyps
5. no family history of CRC,
6. not in compliance with CRC screening guidelines (defined as no sigmoidoscopy within the last 5 years, no colonoscopy within the last 10 years, or no FIT/FOBT within the past year), and
7. not enrolled in any other CRC screening interventions at the time of the study.

**Fig.1:**
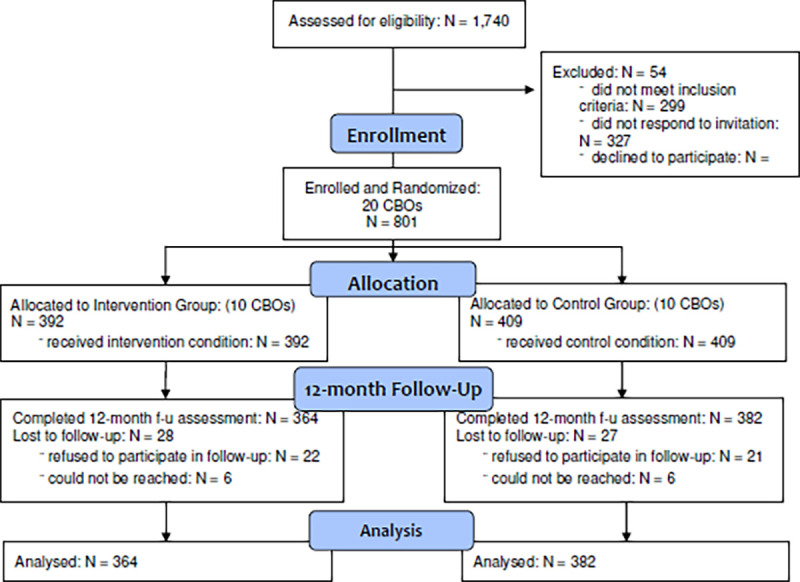
Consolidated Standards of Reporting Trials (CONSORT) Flow Diagram

Our recruitment efforts reached a total of 1,740 Vietnamese American men and women aged 50 or older and residing in the Greater Philadelphia area encompassed by the 20 participating CBOs. Among participants, 1,441 met the inclusion criteria, among which, 801 consented and completed the baseline assessment. Specifically, 392 participants were in the intervention group, while 409 were in the control group. Of the 801 participants who completed the baseline assessment, 746 completed the 12-month follow-up assessment and were included in the analysis sample (intervention group: n = 364; control group: n = 382). Meanwhile, 55 individuals dropped out of the study by the time of follow-up (28 in the intervention group and 27 in the control group). The study retention rate was 93% among participants who completed baseline, intervention, and 12-month follow-up (Figure 1). Informed consent was obtained from all participants in their preferred language, both written and verbal. The study was approved by the Temple University Institutional Review Board (IRB).

### Intervention Components

**Fig. 2:**
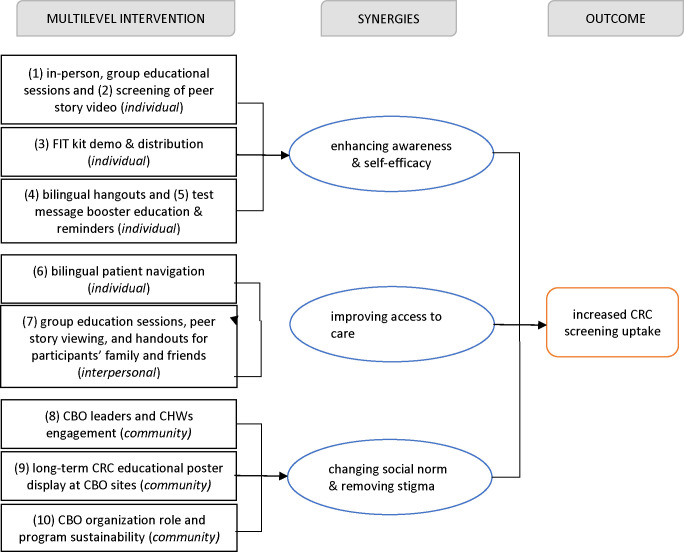
Illustration of how the multilevel intervention components improved CRC screening uptake.

We used the social-ecological perspective [22] and the social cognitive theory [23] as the conceptual framework to guide the design of our intervention. Our intervention included five components to address barriers to CRC screening at the individual, interpersonal, and community level. Figure 2, adapted from previous research [24], depicts the multicomponent and the synergies of our multilevel intervention.

At the individual level, we delivered (1) in-person group-based education to participants in the intervention group at the participating CBO sites. We developed and culturally tailored educational content based on the materials and guidelines from the Centers for Disease Control and Prevention (CDC) and the American Cancer Society. The content included information on definitions, symptoms, epidemiology, protective factors, and risk factors of CRC; information on CRC disparity among Vietnamese Americans; information on the benefits of CRC screening and early detection; and information about tests used to screen for CRC. The information was translated into Vietnamese by certified translators and was reviewed and approved by bilingual community health educators, community leaders, researchers, and a bilingual gastroenterologist. The educational materials were then delivered by bilingual community health workers (CHWs) and patient navigators. Prior to the group education, CHWs underwent a two-day training session, in which they were presented with information about the program, about IRB/human subject protection, and about CRC and CRC screening and in which they received instruction on how to deliver group education, which was reinforced via role-playing and practice.

(2) a peer personal story video on the benefits of CRC screening and early detection. The video was produced by our team in collaboration with community leaders, a bilingual gastroenterologist, and a community peer who had life experience with CRC and who had benefited from CRC screening and early treatment. At the end of the education session, participants were (3) provided with a FIT kit, and bilingual CHWs instructed participants about how to conduct a FIT, using peanut butter as a demo tool [25]. The original English instructions and Vietnamese translations and a return envelope were provided to participants. CHWs answered questions from participants on site and later via email and phone. In addition, (4) bilingual educational handouts were provided to participants. The handouts covered information about colonoscopy preparation, CRC facts, and how to talk to a physician about CRC screening. (5) After group education, participants received five text messages over a five-week period. The text messages included “quick facts” about CRC symptoms and screening guidelines, as well as reminders about setting up appointments with a healthcare provider for CRC screening.

An additional individual-level intervention component was (6) bilingual patient navigation. Bilingual CHWs with strong ties to the community and to the CBOs assisted with various aspects of patient navigation. CHWs coordinated the collection of FIT kit samples from participants and provided further assistance to participants on FIT self-sampling. CHWs also aided participants with abnormal FIT results, including guidance on making appointments for subsequent physician consultation and colonoscopy, if needed.

At the interpersonal level, (7) we encouraged participants to invite family members or friends to group education sessions. The peer personal story video, which was posted on a public-facing website set up for the research project, was shared with participants’ family members and friends.. Educational brochures on CRC were provided to participants’ family members and friends following group education.

At the community-organizational level, we reduced stigma related to CRC screening by cultivating a safe and open space and supportive environment for preventive health behaviors. We designed community organizational-level intervention components. These components served an essential role in facilitating positive changes in organizational roles of the CBO and in community-wide social norms. The research team engaged CBO leaders and community health workers (8) culturally tailored education on CRC screening, health promotion, and communication. The training components included in-person educational seminars and handouts, as well as group discussion sessions, and (9) CRC screening promotion via posters and handouts in Vietnamese language to be displayed in CBO conference rooms or lobby areas. The posters and handouts included key information about CRC, screening tests, colonoscopy preparation, and available healthcare resources, including patient navigator contact details. Another component of the organizational-level intervention aimed to improve the long-term role of CBOs and to engage CBOs in promoting CRC screening and other preventive health behaviors within the community.

To ensure the program’s sustainability, CBO leaders and CHWs in the intervention groups received technical assistance to develop and maintain best practice. This included strategies such as opening group education sessions with remarks on CRC disparities among Vietnamese Americans, training in community health navigation, and enhancing CRC screening promotion efforts. These practices were encouraged at regular meetings and at cultural gatherings and events of CBOs. Additionally, CBO leaders adopted the practice for sending CRC screening text reminder messages to every CBO member on their 50^th^ birthday. The text reminder message was adapted from the five text messages received by the study participants in the intervention group.

### Control Condition

Participants in the control group were provided with a group-based education session in a format that was similar to that of the intervention group. The group session focused on general cancer education and preventive care, including the importance of receiving routine health exams and various types of cancer screening. The educational content was based on the CDC’s Clinical Preventive Services (CPS) Guidelines for adults aged 50 and older [26]. Participants were encouraged to schedule routine medical check-ups with their health care providers. All educational workshops were conducted in Vietnamese by bilingual community health educators. The educational content was translated into Vietnamese by certified translators.

### CBPR-based Strategies

The creation of a CBPR environment that is conducive to co-learning and empowering, one that supports, encourages, and enhances the formation of trusting and long-lasting partnerships, is an important strategy in engaging communities in health disparities research [27–29]. We used CBPR-based strategies in multiple aspects of the study design and implementation. These strategies underscore several aspects of CBPR, including community involvement in recruitment of study sites and participants, intervention design, and data collection. The community engagement was a heuristic, non-linear process, with the different aspects interwoven. Each aspect is detailed below.

### Community-Engaged Recruitment: CBO Sites and Participants

Community leaders influential in the Vietnamese American community in the Greater Philadelphia area served as advisors of the Community Advisory Board (CAB) to this project who closely involved in all aspects of the study. The contributions of the CAB and leaders of collaborating CBO sites toward the intervention planning and implementation were substantial. Specifically, they played a key role in the design of the intervention and educational messages by providing community-specific information and insight on participant recruitment strategies, health beliefs and cultural issues that proved relevant in the design of culturally sensitive messages and program delivery approaches, and research data collection instruments.

In the context of this study, a site or cluster was a specific CBO in the Vietnamese American communities. The CBOs included churches, temples, adult daycare centers, community centers, and other types of community associations. Site recruitment was built on relationships between the research team and Vietnamese American community leaders and stakeholders that were established previously through decades-long collaborative research experience and community engagement. New relationships were also brought to the project through more recent outreach. Several church pastors and CHWs played significant roles in the recruitment of sites. They were actively involved in identifying sites that could be included in the study and in connecting the research team with other organizations that could be appropriate targets for recruitment. They further served as a liaison between the research team and the community, facilitating bi-directional communication on study purpose, design, and implementation. Their role in establishing a shared mission and trust between the research team and the CBOs was crucial to the success of site recruitment.

Community liaisons served a similar role in the recruitment of participants within CBOs. They provided input on the content design and distribution of recruitment flyers, ensuring that the messages, in both English and Vietnamese, were culturally sensitive and accessible to community members. Their active roles in communication with potential study participants were also significant. Notably, they joined the research staff in explaining the research purpose and protocol to the community, and they answered questions and addressed concerns from potential participants. They also participated in regular program planning meetings, providing insightful comments on recruitment strategies, recruitment feasibility, and potential demographic composition of the participants in specific sites.

### Community Involvement in the Development and Dissemination of Culturally Tailored Education Materials

We adapted educational curricula from prior studies effective in raising cancer awareness and screening uptake in Asian and minority populations [28, 30–32]. We incorporated facts and guidelines on CRC prevention and screening tools, CRC management from the National Cancer Institute, (cancer.gov), the Centers for Disease Control and Prevention (CDC.gov), the Mayo Clinic, (MayoClinic.org), and the American Cancer Society (Cancer.org). The curriculum used in the education sessions included 8 modules that explained cancer and CRC, CRC burdens in Vietnamese Americans, symptoms and risk factors of CRC, CRC screening and early detection methods, stages of CRC, treatment of CRC, and common misconceptions and myths about CRC. Handouts included key facts and infographics on CRC and CRC screening, in both English and Vietnamese. The CBO leaders, and CHWs were actively engaged in the design of not only the content of the educational curriculum but also the format of education workshop delivery. CBO leaders and CHWs contributed to the curriculum’s content and format, ensuring cultural relevance, accessibility, and linguistic appropriateness. They also guided the research team in delivering education sessions, which involved CHWs leading the sessions, group viewing of peer story videos, and subsequent discussions.

### Community engagement in data collection

The community leaders and CHWs provided feedback on the survey questionnaires used for program evaluation. They gave comments and suggestions on draft versions of the surveys, specifically on the accessibility of the language used in the survey questions, length of the questionnaire, relevance of the measures included, and appropriateness of specific wordings used. In addition, they played crucial roles in organizing and conducting in-person and online data collection for baseline and 12-month follow-up assessments with the participants and surveys with family members, friends, and CBO leaders. They offered logistical assistance in in-person data collection efforts, language assistance to the participants in completing the surveys, and coordination for follow-up telephone survey outreach to participants, family members and friends, and CBO leaders.

### Data collection

#### Individual Level:

The baseline surveys were conducted in person at the CBO sites, taking approximately 20 minutes, while the 12-month follow-ups were completed via phone, lasting around 10 minutes. Participants had the option of answering the questions in either English or Vietnamese for both baseline and 12-month follow-up surveys. Onsite language assistance was available for participants, being provided by a trained bilingual community health educator. Primary outcome data on the uptake of FIT testing was collected objectively. Following the receipt of second-generation FIT kits (Fecal Immunochemical Test), participants in the intervention group used the kit to collect a small sample of stool at home and results were ready within five minutes. Specifically, participants unscrewed the collection tube to expose the wand and collected a small sample of stool, then inserted the wand back into the tube, unscrewed the smaller cap and added three drops of solution to the cassette. Available results’ images were uploaded and sent to the study team via mobile devices. Participants with a negative result were considered screened for colon cancer and recommended to test again in a year, while a positive result were navigated to a physician for a follow up of colonoscopy.

#### Interpersonal Level:

We also surveyed 60 family members or friends of the participants, including 30 family members or friends of participants in the intervention group and 30 in the control group. As we conducted the 12-month follow-up survey, we asked each participant to recommend a family member or peer who had participated in the group education sessions, had participated in peer story video viewing, had received any handouts about CRC or CRC screening, had been in a conversation with the participant about CRC screening, or had influenced the participant’s decision about whether or not to undergo CRC screening. With contact information provided by participants, the research team reached out to family members or friends to complete the surveys via telephone.

#### Community-Organizational Level:

During the 12-month follow-up surveys with participants, we also surveyed two CBO leaders from each site in-person or via telephone. Each CBO leader survey lasted 10 minutes. We surveyed 40 CBO leaders from the 20 CBO study sites, asking them to assess their CBOs’ engagement in various aspects of the project and their perception of the social norms related to CRC and CRC screening.

### Measurement

Outcome variables of the study included the uptake of any CRC tests and the uptake of FIT at the 12-month follow-up survey. Participants were asked if they had any tests for CRC over the past 12 months, including whether they had a FIT. Verification of FIT test was obtained as described above. If any participants indicated the uptake of colonoscopy, verification was requested.

Gender was assessed with two categories (male or female), and age was assessed with three categories (50–64, 65–74, or 75 and above). Educational attainment was measured as the highest level of degree obtained. The responses were coded as 0 for “high school degree or lower” and 1 for “ college or advanced degree.” English proficiency was measured in two categories, “not at all or not well” or “well or very well.” Health insurance coverage was measured with the question: “do you currently have health insurance?” The responses were coded as 0 for “no insurance” and 1 for “yes.”

We assessed participants’ CRC-related knowledge with 9 questions. The questions covered a variety of areas regarding general knowledge of CRC and how it relates to the Vietnamese American population, including the appropriate age to begin CRC screening, risk factors for CRC, and the severity of risk for Vietnamese populations in the United States. A composite score from 0 to 9 was calculated from responses, with higher numeric value indicating higher level of CRC Knowledge.

We also examined two CBO site-level measures: social norm and time of 12-month data collection. We computed the CBO-level social norm score with 8 community-based questions related to CRC screening from the surveys with the CBO leaders. The goal was to obtain information on communal views of CRC screening, which may have influenced previous screening uptake. Social norm questions were asked using a 5-point Likert scale, ranging from 0 for “strongly disagree” to 4 for “strongly agree.” The questions included “people in my community talk about colorectal cancer screening,” “people in my community believe colorectal cancer screening can help prevent colorectal cancer,” and “talking about colorectal cancer and colorectal cancer screening is a comfortable topic in my community.” The average score was calculated for the 8 questions, ranging from 0 to 24, with a higher numeric value indicating a greater presence of positive social norm toward CRC and CRC screening. We then took the average of the scores to generate a social norm score for the given CBO site. In addition, we examined whether the data collection of the 12-month assessment for a given CBO site was conducted before or after the COVID-19 outbreak (March 2020), given that the pandemic might have affected participants’ access to CRC screening, intention to undergo screening, and other relevant factors.

### Statistical Analysis

To test the differences in sociodemographic and health-related factors between the two study arms, we conducted chi-square tests for categorical variables and t-tests for continuous variables. We fitted two generalized linear mixed-effect models for each of the outcome variables to examine the intervention effects: the first model without any covariates, and the second model accounting for age, gender, health insurance coverage, English proficiency, CRC-related knowledge, site-level CRC-related social norm, and time of 12-month follow-up assessment. We also examined attitudes and behaviors related to CRC screening and provision of support for CRC screening uptake among the participants’ family members and friends. For the CBO sites in the intervention group, we examined the CBO leaders’ assessment of the partnership between the CBO and the research team. Program evaluation data from the 12-month follow-up survey was also analyzed. We conducted all analyses for this study in Stata 16 [33].

## Results

The baseline characteristics of the participants in the intervention and control groups, along with time of the 12-month data collection, are presented in Table 1. The 2 groups were comparable regarding sex, age, education level, and English proficiency. However, the intervention group had a higher proportion of uninsured individuals (14.7%) than did the control group (8.9%). CRC-related knowledge scores and social norm scores also varied significantly between the two study arms. Furthermore, a higher proportion of CBO sites in the control group collected data before the COVID-19 outbreak.

Table 2 presents the generalized linear mixed-effect model (OR and 95% confidence intervals [CIs]) for the primary study outcome, controlling for age, gender, health insurance coverage, English proficiency, CRC-related knowledge, CRC-related social norm, and collection of data before or after the COVID-19 outbreak. The intervention group had a substantially higher CRC screening rate at follow-up than did the control group (91.48% vs 42.41%). In the generalized linear mixed-effect model, the ORs of participants receiving CRC screening by 12-month follow-up were significantly higher for the intervention group than for the control group (OR= 34.61; 95% CI = 14.70 – 81.52; p = 0.05). After adjusting for covariables, the OR of receiving CRC screening between the intervention and the control group increased to 49.27 (95% CI = 17.65 – 137.56; p = 0.05).

Table 3 presents the generalized linear mixed-effect model of FIT uptake at 12-month follow-up. The intervention group had a significantly higher FIT uptake rate at follow-up than did the control group (89.56% vs 7.59%). In the generalized linear mixed-effect model, the ORs of participants with FIT uptake by 12-month follow-up were significantly higher for the intervention group than for the control group (OR= 4028.63; 95% CI = 401.09 – 40464.48; p = 0.05). After adjusting for covariables, the OR of receiving CRC screening between the intervention and the control group decreased to 377.72 (95% CI = 376.95 – 378.49; p = 0.05).

**Fig. 3:**
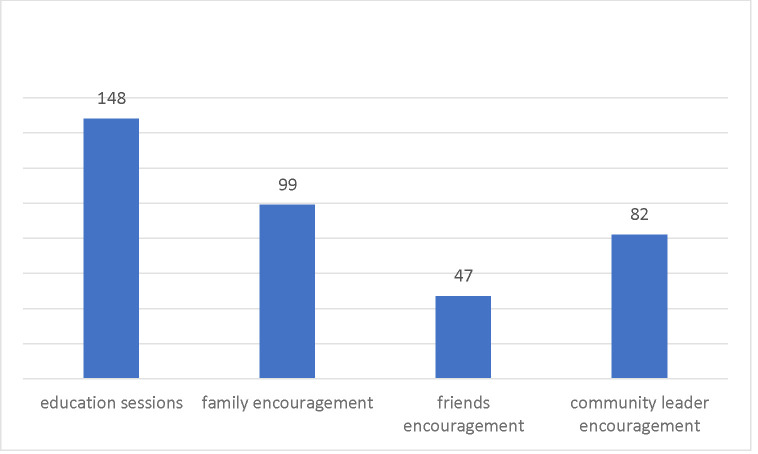
Motivating Factors for CRC Testing Among Individuals from the Intervention Group (N = 326)

Figure 3 shows the motivating factors for CRC testing uptake. Among the 326 individuals in the intervention group who reported receiving CRC testing at the 12-month follow-up assessment, 148 (45.40%) reported that information received from the education sessions motivated them to undergo testing for CRC. In addition, 99 participants (30.37%) reported encouragement from family members as a factor, 47 (14.42%) reported encouragement from friends as a factor, and 82 (25.15%) cited encouragement from community leaders as a motivating factor for CRC testing uptake.

**Figure 4.**
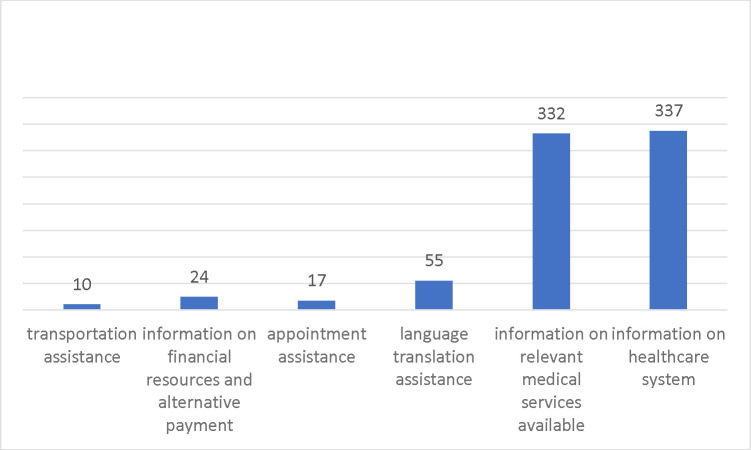
Patient Navigation Received by Individuals in the Intervention Group (N = 364)

In addition, we examined the various types of patient navigation received by participants in the intervention group (Figure 4). A very high proportion of participants reported receiving information regarding relevant medical services (332, 91.21%) and receiving information about the healthcare system in general (337 92.58%). In addition, 55 (15.11%) reported receiving language translation assistance. Less than 10% of participants reported receiving assistance with information on financial resources, making appointments, or transportation.

## Discussion

When detected at an early, localized stage (stages I and II), the 5-year survival rate of CRC approaches 90% [34]. By comparison, 5-year survival for those diagnosed with late-stage CRC (stage IV) is below 15% [34]. Despite the well-known benefits of CRC screening and early detection, screening uptake remains suboptimal in the general US population and is noticeably lower among racial/ethnic minorities, especially among those without health insurance, with low socioeconomic status, and with limited English proficiency, , including underserved Vietnamese and other Asian Americans [35, 36]. Suboptimal CRC screening in these groups falls far short of the targets set by the Healthy People 2030 initiative [12]. Inexpensive non-invasive fecal CRC tests, such as FIT and gFOBT, offer a cost-effective way for improving population-based screening programs, especially in low-resource settings and among low-income immigrant populations [37, 38].

As demonstrated in this study, our community-based multilevel intervention *had significant effects* on CRC screening uptake among Vietnamese Americans over age 50. The high FIT screening rates achieved in the intervention group (89.56%) are attributed to the combined effects of multilevel intervention components, including individual-level education and patient navigation, interpersonal-level support, and community-level destigmatization and changes in social norms. Similar measures have been shown to be effective in increasing screening uptake for CRC, other types of cancer, and cancer-related diseases among Vietnamese communities [8, 16, 20, 29, 39, 40]. Educational information, provided in multiple formats, was heavily cited by participants in the intervention group as a factor that motivated them to obtain CRC screening. Furthermore, participants noted that encouragement from family members, community leaders, and, to a slightly lesser extent, friends, was important in their decision to undergo CRC screening. Our findings also highlight the significance of engaging community leaders, CHWs, health providers providing culturally responsive care, and other community stakeholders in the design, dissemination, and implementation of the interventions. Their engagement in the research implementation process was dynamic, with their roles naturally evolving as the study progressed.

However, our recruitment faced significant challenges, primarily due to low awareness and stigma surrounding CRC in the community. Many community members and CBOs either underestimated CRC’s relevance or were reluctant to discuss it outside family circles. To address this issue, we created concise “quick fact” sheets and informative materials to educate and engage CBO leaders and staff. Understanding the importance of CRC screening was crucial for effective community outreach and recruitment. Once we ensured the CBOs leaders’ commitment to the issue, we collaborated with them to initiate recruitment among community members. Equipped with more knowledge of CRC and CRC screening, leveraging the trust and the respect we established in the community, the CBO leaders and study team members were able to better communicate to community members, gauge their interests, and address their concerns. CBO leaders and research team ensured that our outreach channels were appropriate and that the language we used was accessible to the target population. Bilingual infographics and brochures that the research team developed together with CBO leaders were effective tools in facilitating communication in the recruitment process.

Another challenge we faced was fear caused by the federal government’s public charge rule, which went into effect on February 4, 2020. The public charge rule essentially counted the use of certain health or human services as a negative factor in immigration application review. Even though the law applied the public charge test only to a small segment of the immigrant population, it created fear and included the “chilling effect” wherein immigrants chose to avoid using public benefits or any health services due to worries about legal sanctions. Although our community patient navigators, together with CBO leaders and research team, provided clarification and reassurance of participant confidentiality in the study, the chilling effect was nonetheless observed [41].

Our study was not without limitations. First, the two study arms differed in several sociodemographic characteristics. Notably, the intervention group had a higher proportion of individuals who lacked health insurance, a lower average score for CRC-related social norm, and a higher proportion of CBO sites where 12-month data collection was conducted after the COVID-19 outbreak in March 2020. We controlled these factors in the regression analysis. Second, we were unable to determine the independent effects of individual program components on the outcome measures. A stepwise design should be considered for future interventions to better identify the efficacy of individual components in a multilevel intervention.

In conclusion, our study used community-based participatory research (CBPR) approach to address colorectal cancer disparities experienced by underserved Vietnamese Americans. Our study findings demonstrated significant evidence that community-based multilevel interventions with culturally tailored components are effective for addressing healthcare access barriers and for promoting CRC screening uptake among medically underserved Vietnamese Americans. Future efforts should focus on adapting, scaling up the evidence-based intervention for implementation in other Asian Americans and racial/ethnic populations affected by CRC disparities. Our study marks a pivotal step forward in promoting CRC screening uptake via FIT, a noninvasive and cost-effective screening tool. By grounding our efforts in community partnerships through trust and capacity building, power sharing and organizational/system change, our study has significant implications for broad program implementation aimed at reducing CRC health disparities.

## Figures and Tables

**Table 1.** Baseline Characteristics of the Participants and Time of Site 12-Month Data Collection by Study Arm and In Total (N = 746)

	Total (N=746)	Intervention (N=364)	Control (N=382)	p-value
Gender				0.095
male	309 (41.4%)	162 (44.5%)	147 (38.5%)	
female	437 (58.6%)	202 (55.5%)	235 (61.5%)	
Age				0.163
50–64	365 (48.9%)	191 (52.5%)	174 (45.5%)	
65–74	253 (33.9%)	116 (31.9%)	137 (35.9%)	
75+	128 (17.2%)	57 (15.7%)	71 (18.6%)	
Education level				0.096
<= high school	591 (79.2%)	294 (84.0%)	297 (79.2%)	
college+	134 (18.0%)	56 (16.0%)	78 (20.8%)	
English proficiency				0.475
not at all or not well	687 (94.9%)	330 (94.3%)	357 (95.5%)	
well or very well	37 (5.1)	20 (5.7%)	17 (4.5%)	
Health insurance				0.019
no insurance	78 (11.7 %)	47 (14.7%)	31 (8.9%)	
yes	590 (88.3%)	272 (85.3%)	318 (91.1%)	
CRC-related knowledge score				< 0.001
Mean (SD)	3.828 (1.156)	3.984 (1.093)	3.681 (1.196)	
Range	0.000 – 9.000	0.000 – 9.000	0.000 – 9.000	
CRC-related social norm score (Site-Level)				< 0.001
Mean (SD)	16.572 (2.312)	16.048 (1.515)	17.071 (2.784)	
Range	14 – 23	14 – 19	14 – 23	
Time of 12-Month Data Collection (Site-Level)				< 0.001
data collected before March 2020 (COVID-19 outbreak)	560 (75.1%)	248 (68.1%)	312 (81.7%)	
data collected after March 2020	186 (24.9%)	116 (31.9%)	70 (18.3%)	

Note: P-values presented in this table were results from chi-square test for categorical variables and student’s t-test for continuous variables.

**Table 2.** Generalized Linear Mixed-Effect Model of Having Any CRC Screening Tests Done at 12-Month Follow-Up Among Low-Income Vietnamese Americans Over Age 50 (N = 746)

Variable	Total	Intervention	Control
No. clusters	23	11	12
No. participants	746	364	382
Any CRC screening test, %	66.35%	91.48%	42.41%
Model 1, OR (95% CI)		34.61 (14.70 – 81.52)	1 (Ref)
Model 2, OR (95% CI)		49.27 (17.65 – 137.56)	1 (Ref)

Abbreviations: OR = odds ratio; CI = confidence interval

Note: Model 1 did not adjust for any covariates. Model 2 adjusted for age, gender, health insurance coverage, English proficiency, CRC-related knowledge, CRC-related social norm, and whether data was collected before or after the COVID-19 outbreak.

**Table 3.** Generalized Linear Mixed-Effect Model of FIT Uptake at 12-Month Follow-Up Among Low-Income Vietnamese Americans Over Age 50 (N = 746)

Variable	Total	Intervention	Control
No. Clusters	23	11	12
No. participants	746	364	382
Done FIT test, %	47.59%	89.56%	7.59%
Model 1, OR (95% CI)		4028.63 (401.09 – 40464.48)	1 (Ref)
Model 2, OR (95% CI)		377.72 (376.95 – 378.49)	1 (Ref)

Abbreviations: OR = odds ratio; CI = confidence interval

Note: Model 1 did not adjust for any covariates. Model 2 adjusted for age, gender, health insurance coverage, English proficiency, CRC-related knowledge, CRC-related social norm, and whether the data was collected before or after the COVID-19 outbreak.

## Data Availability

To ensure participants’ confidentiality, the data of this study is not publicly available.
